# The Week's Work

**Published:** 1911-04-29

**Authors:** 


### The Week's Work.

### MR. PERCEVAL A. NAIRNE.

Last week we published in the "Eminent Chairmen" series an article upon Mr. Nairne, the very able and influential chairman of the Dreadnought Seaman's Hospital. There has been evidence of friendly rivalry for public recognition amongst a few men in the hospital world, in recent years especially. Men of Mr. Nairne's type are never found in such an arena. They take up work for the hospitals as a part of their daily life, they love the work, and they are contented to do it to the utmost of their ability, unrecognised by, and often unknown to the world outside the particular hospital they may control. Yet it is probably true to say that no hospital chairman of to-day carries with him in reality greater authority or influence than Mr. Nairne. The nation owes the Tropical School of Medicine largely to the courage and firmness exhibited by this gentleman in the face of much opposition. This school is greatly indebted to Mr. Joseph Chamberlain, who at the time of its inception was Secretary of State for the Colonies, and the initial steps were taken at the Colonial Office. There was formed a large and influential advisory committee comprising the leaders of the medical profession, Colonial governors, and other representative men, which laid the foundations of the new school. This committee, of which Mr. Nairne was chairman, who remains still the chairman of the School Committee, carried through the whole of the negotiations which eventuated in the Tropical School of Medicine. Originally constructed for twenty students only, the school now contains accommodation for upwards of sixty, and every year upwards of a hundred graduates pass through its course. In this, the Coronation year of King George, who has earned the title of the Hospital King, it would be both welcome and popular to find that work such as that done by Mr. Nairne is not overlooked when the honours' list appears on Coronation Day.

### A PERFECTED WARD UNIT.

It is always our wish to make The Hospital of practical assistance to all who are engaged in the management of a modern hospital. Having this in view, we invited the co-operation of a few of the leading hospital architects, who were each asked to prepare a plan of a complete ward unit for a modern hospital. We have now the pleasure to announce that next week we propose to publish the first plan of such a perfected unit, with the grounds on which the authors have designed and now recommend it as a good representative type of what a modern ward, planned in the completest and best way, should in fact be. We are confident that these articles will excite a good deal of interest, and we hope that interest may crystallise in criticism and suggestion. If so, we shall be glad to open our columns, and to encourage a full discussion of every point of practical interest which may be involved.

### THE DONCASTER ROYAL INFIRMARY'S RECONSTRUCTION SCHEME.

From the report, which we publish on another page, of the desperate attempts which the Doncaster Royal Infirmary has made to meet a situation of the gravest difficulty, it would seem as if the hospital's committee had an unanswerable case for the reconstruction on which it decided six months ago. The want of accommodation, the expedients adopted to supply it, the inadaptability of the present site, the growing colliery population, and the increased wealth consequent on this, form a combination of circumstances that would appear certain to enlist public support. Nevertheless, the fact remains that the New Hospital Fund has been virtually a failure, for £888 in six months is not success. No doubt the hospital authorities have been occupied so fully with the internal rearrangements which have increased the number of beds, that they have had time for little else. But the surprising feature in the situation really is that no one of private or municipal standing seems to have grasped the position or to have put himself at the head of a public movement on the Infirmary's behalf. Up to the present Doncaster has taken no step to commemorate King Edward VII. That being so, the infirmary of which he was a life governor has not unnaturally been passed by. Surely, however, this indifference cannot continue. An appeal should be made immediately, with the co-operation of the Mayors of Doncaster and the boroughs, to remove the slur that must remain on any town without a King Edward memorial, and to testify publicly to the enterprise of the hospital's recent expedients by making the New Hospital Fund a great success. Will no two rich men in the neighbourhood promise £10,000 as a start? We believe that energy on the part of the Mayors could raise a similar amount before the Coronation is over.

### HOSPITALS, PRACTITIONERS, AND THE POOR LAW.

In the instructive speech which we reported last week, it is noteworthy that Sir Savile Crossley at the recent meeting of the Hospital Saturday Fund emphasised the relation of the three health agencies mentioned above. He repeated the old fact that is still in need of emphasis, that the voluntary hospitals are intended for a special class, the class that is able in times of health to pay for itself, but which is unable to stand the strain of illness. "If they are not able to pay anything at all, they are Poor Law cases." Again, the hospitals are places of consultation where may be brought those cases which the local practitioner has failed, or should not attempt, to cure. There is little startling in these words, but we quote them here to show the increasing tendency to couple together in a great scheme of co-operation all existing forms of sick relief. It is in the conscious relation of the Poor Law infirmary to the voluntary hospital that the State interference of the future must lie. When the out-patient department has been replaced by casualty and consulting rooms, the private practitioner will cease to find the hospital a competitor. It is important to remember that this scheme for bringing the three health agencies into co-operation involves no large expenditure, like, for instance, the municipalisation proposals. It is a reform which can be brought about by relatively simple changes in administration, and it is the only plan yet offered which does not sacrifice the existence of any of the three agencies concerned.

### THE NEW PURCHASE OF LAND FOR LEEDS INFIRMARY.

On Tuesday last week Leeds General Infirmary became the possessor of a very considerable addition of land, on which the extensions that were decided upon as the result of Sir Henry Burdett's report will be carried out. The new land, which covers an area of 8,400 square yards, has been purchased for £17,000, and by it the boundary line of the Infirmary is moved from Thoresby Street to the north of Sunny Bank, where a new road will be cut, linking up S. James Street and Fenton Street. This is the briefest intelligible summary that can be given in words of the extension, and is all that it is advisable to say until the detailed plans for dealing with the new area have been decided on. But, speaking generally, we may again remind our readers that four new theatres will be built, at a cost of about £12,000; that accommodation for thirty more patients will be provided at a cost of £5,000; that ten wards are to be modernised, at a cost of £700 each, and that some £4,000 is to be spent in alterations to the roofs of the Infirmary, whereby all risks of fire may be avoided. It will be remembered that a fire last October destroyed an important part of the east wing, and so no one will grudge this item in the expenditure. The fact that the purchase of this additional land has been completed should give a great stimulus to the hospital's friends in Leeds, as showing that the town's memorial to King Edward is being enthusiastically carried. The sum wanted is £150,000; as we go to press we learn that two-thirds of this have been subscribed.

### THE LATEST HOSPITAL SCANDAL.

On Monday last the afternoon papers announced in huge letters on their placards the occurrence of "shocking and amazing things at a hospital." The well-known vagueness that is the ideal for which the sub-editor strives in designing these contents bills probably accounted for the eager rush on the part of the public to read the details of this scandal. A perusal of the few facts related showed very clearly, however, that once again the desire to make your flesh creep, which is as rampant in the lay Press when hospital matters are concerned as it was in The Fat Boy, had led to an exaggeration of the importance of the facts. Briefly put, the scandal resolved itself into this. At an inquest at the Westminster Coroner's Court, held on Monday last, it was found that the deceased had been operated on in St. George's Hospital for what was presumably spinal caries. While under the influence of the anæsthetic and being carried back to the ward on a water-bed, the porters carrying the stretcher allowed it to slip down, with the result that the patient fell on to the floor. This accident, it is amply proved, had nothing whatever to do with the patient's death some weeks subsequently, which was directly due to tuberculous meningitis. The jury, in finding a verdict in accordance with the facts, "deplored the neglect of those concerned in not taking proper precautions in moving the patient." In reply to the legal representative of the hospital, who attended the inquest, the foreman stated that the jury regarded the fall as purely accidental, and had no wish to cast any reflection on the authorities, and finally the word "neglect" was withdrawn.

### SOME REFLECTIONS.

The medical evidence made it abundantly clear that there was no direct relation between the accident and the patient's death. The fall occurred on February 4 last, and the patient died on April 20. At the same time it is exceedingly difficult to dismiss wholly the suggestion that the fall had no indirect relation to the fatal result. We have on more than one occasion drawn attention to the antiquated methods of moving patients, and particularly narcotised patients, from and to the wards. The proper way in which such patients should be moved is on a pneumatic or rubber-tyred stretcher, which can be quietly wheeled through the corridors and wards without disturbing other patients, or the slightest risk or discomfort to the anæsthetised patient himself. To lift and carry such patients on the old-fashioned hand stretchers is to risk such an unfortunate accident as happened at St. George's. In the case of a child there may be reasons for carrying the little patient in the arms, but in the case of an adult the possibility of a fall should be absolutely eliminated by the use of strongly-made wheeled stretchers.

### THIS WEEK'S DRUG MARKET.

Business continues quiet and few changes in price of importance have taken place during the week. As was not unexpected, importers of quicksilver have reduced the price and a reduction in the price of calomel and other salts of mercury may possibly be made by the makers. Opium is still tending dearer, as also is morphine. The demand for cod-liver oil continues very quiet and the tendency of prices is in a downward direction. Ipecacuanha is rather dearer.

